# Could Causal Discovery in Proteogenomics Assist in Understanding Gene–Protein Relations? A Perennial Fruit Tree Case Study Using Sweet Cherry as a Model

**DOI:** 10.3390/cells11010092

**Published:** 2021-12-29

**Authors:** Maria Ganopoulou, Michail Michailidis, Lefteris Angelis, Ioannis Ganopoulos, Athanassios Molassiotis, Aliki Xanthopoulou, Theodoros Moysiadis

**Affiliations:** 1 School of Informatics, Aristotle University of Thessaloniki, 54124 Thessaloniki, Greece; lef@csd.auth.gr; 2 Laboratory of Pomology, Department of Horticulture, Aristotle University of Thessaloniki, Thermi, 57001 Thessaloniki, Greece; msmichai@agro.auth.gr (M.M.); amolasio@agro.auth.gr (A.M.); aliki.xanthopoulou@gmail.com (A.X.); 3 Institute of Plant Breeding and Genetic Resources, ELGO-DIMITRA, Thermi, 57001 Thessaloniki, Greece; giannis.ganopoulos@gmail.com; 4 Department of Computer Science, School of Sciences and Engineering, University of Nicosia, Nicosia 2417, Cyprus

**Keywords:** causality, DAG, PC algorithm, proteogenomics, sweet cherry, WGCNA

## Abstract

Genome-wide transcriptome analysis is a method that produces important data on plant biology at a systemic level. The lack of understanding of the relationships between proteins and genes in plants necessitates a further thorough analysis at the proteogenomic level. Recently, our group generated a quantitative proteogenomic atlas of 15 sweet cherry (*Prunus avium* L.) cv. ‘Tragana Edessis’ tissues represented by 29,247 genes and 7584 proteins. The aim of the current study was to perform a targeted analysis at the gene/protein level to assess the structure of their relation, and the biological implications. Weighted correlation network analysis and causal modeling were employed to, respectively, cluster the gene/protein pairs, and reveal their cause–effect relations, aiming to assess the associated biological functions. To the best of our knowledge, this is the first time that causal modeling has been employed within the proteogenomics concept in plants. The analysis revealed the complex nature of causal relations among genes/proteins that are important for traits of interest in perennial fruit trees, particularly regarding the fruit softening and ripening process in sweet cherry. Causal discovery could be used to highlight persistent relations at the gene/protein level, stimulating biological interpretation and facilitating further study of the proteogenomic atlas in plants.

## 1. Introduction

Among the numerous research areas of biology, the interactions of proteins and genes of an organism as well as the expression of genes and proteins are a topic of paramount importance [1]. A popular method that aims to analyze the diversity of different biological samples is large-scale transcriptome profiling. Most transcriptome analyses focus on specific organs or entire organizations, such as plants. Moreover, new trends in research aiming at better understanding of gene function, have lead to an undiminished interest in the study of transcriptome profiles of specific tissues or cells [2].

Proteogenomics is a new approach that opens new horizons in the analysis of proteomic and genomic data [3]. The goal of proteogenomic analysis is to combine changes at the protein level with changes at the genetic level (e.g., mutations, polymorphisms, insertions/deletions) [4]. Proteogenomic databases essentially use transcriptional and proteomic data to link gene expression to proteins in order to further understand gene models [3,5,6]. A worthwhile application of proteogenomics in humans resulted in the creation of a global expression atlas that revealed gene/protein expression data in different tissues [7,8]. Recently, in the context of the Human Protein Atlas (HPA) project, a comprehensive map of transcripts and proteins of a plethora of healthy human tissues (18,072 transcripts and 13,640 proteins) was created [9]. The development of a plant tissue atlas containing a combination of transcriptomic and proteomic data has been recently reported in sweet cherry (*Prunus avium* L.) tree [10]. Sweet cherry is a perennial fruit tree of the Rosaceae family, whose economic value occupies a high position in the international economic ranking [11,12,13]. The non-climacteric ripening behavior of sweet cherry fruit are different from several other Prunus species such as peach and apricot, making its study of high interest [14]. Remarkably, cherries are harvested and marketed with their stem, which exhibits tissue specific physiological and metabolic differences to that of the edible fruit part across the whole fruit development and ripening [13]. In addition, the simple sweet cherry genome (2n  =  2x  =  16, genome size of ∼380 Mb) makes it an ideal tree species for the deciphering of various biological phenomena, notably the fruit ripening process [15].

In a recent study [16], the authors used data from both the transcriptome and the proteome to assess and study the changes from a proliferating myeloid progenitor cell in the bone marrow into a mature non-dividing polymorphonuclear blood cell. Based on 2429 transcript–protein pairs that were differentially expressed during the five developmental stages in neutrophil development, they performed weighted gene co-expression network analysis (WGCNA) [17], and identified 12 modules/clusters. In addition, a neutrophil differentiation module network was developed, where modules (network nodes) were pairwise linked with undirected edges when the Pearson correlation coefficient was larger than 0.6. This network revealed that modules with similar functions were connected [16].

Although the Pearson correlation coefficient is a standard approach to assess the association, it has the disadvantage that it can only account for the existence of linear relation. On the other hand, causal discovery justifies the causal nature of an association between two variables on the basis of its persistence [18]. Persistence is the main characteristic of a causal relationship, and the test of a causal relationship involves all other variables of a data set and considers all circumstances [19]. In other words, the causal nature of association is expected to exist in all situations without being affected by the values of other variables. Consequently, the causal relationships tend to be less spurious or volatile than statistical associations, such as correlation [18]. An additional benefit in causal model development is the existence of direction in the causal relations between variables, determining the cause and the effect in each relation [18].

The aim of this study was to employ the dynamics of causal models at a proteogenomic level to in-depth characterize the gene/protein interaction models, notably in the context of sweet cherry fruit development and ripening. The proposed methodological approach initially involved WGCNA to identify the consensus gene/protein modules (clusters). Then, causal discovery was used to evaluate the causal relations among the modules and their associated biological functions. To the best of our knowledge, causal models are used for the first time in this framework and could possibly result in more persistent relations compared to other association measures. Furthermore, the direction of the identified causal relations in the estimated causal network of modules will reflect the cause and the effect in the pairwise module relations. Such an analytical approach may provide valuable insight between functions associated with the gene/protein modules and could reveal new knowledge related to sweet cherry biology, especially in fruit ripening syndrome.

## 2. Materials and Methods

### 2.1. Data Description

Fifteen sweet cherry tissue samples from the cultivar ‘Tragana Edessis’ were collected, covering most organs (leaves, shoot, bud, flowers, stem/pedicle, and fruit) across selected developmental stages, as recently reported [10]. Briefly, we sampled annual sweet cherry shoot (annual shoot), light green leaves (‘young leaves’), mature leaves (‘mature leaves’), flower and vegetative buds (ecodormancy stage), and flowers at both sepal-open stage (‘flower closed’) and full flowering phase (‘flower open’). Sweet cherry fruit (exocarp plus mesocarp) were sampled during four developmental stages (FS) by freezing whole fruit and removing the endocarp. The first stage (8 days after full bloom [DAFB]; 1st fruit stage; FS1 stage) corresponds to the fruit set; the second stage (20d DAFB]; 2nd fruit stage; FS2 stage) was at the beginning of fruit coloring; the third stage (34d DAFB; 3rd fruit stage; FS3 stage) was the coloring advanced; the fourth stage (44d DAFB; 4th fruit stage; FS4 stage) referred to the fruit ripe for harvesting stage (17.5° Brix). Along with fruit, the corresponding stems were collected at the same developmental stages (1–4 stages; SS1–SS4 stages). The transcript and protein expression abundances were retrieved from SweetBiOmics database (www.GrCherrydb.com, data accessed on 1 November 2021).

### 2.2. PC Algorithm

The constrained-based PC algorithm (named after its inventors Peter Spirtes and Clark Glymour, [20,21]) is the method used to learn the causal structure induced by a causal Bayesian network. Particularly, for each pair of variables (X, Y) with a dataset, the PC algorithm assesses their conditional independence given the remaining variables, and it claims the nonexistence of a causal relationship between X and Y, i.e., no edge to be drawn between X and Y in the corresponding graph, when X and Y are independent given some other variables. Essentially, the PC algorithm examines the association of X and Y, conditioning on all subsets of all the remaining variables, in order to determine whether their association is persistent [18]. A relationship is causal when the association exists given each of the conditioning sets. The output of the PC algorithm is a network with a structure consistent with the results of the tests of independence. It is assumed that causal sufficiency holds [21]. Specifically, this condition implies that for every pair of measured variables, all their common direct causes are also measured. In other words, there are no hidden, unmeasured confounders for any pair of variables.

The network is represented by a Markov equivalence class of the Directed Acyclic Graph (DAG). All DAGs in an equivalence class describe the same conditional independence relationships since they have the same skeleton (adjacencies) and the same v-structures. Assume G is a DAG, then the skeleton of G is the undirected graph formed by removing directions of all the edges in the DAG. A v-structure in G is an ordered triplet of nodes (x, y, z), such that G contains the directions of x → y and y ← z, and also the nodes x, z are not connected with an edge in G. However, some edges may have an undetermined direction (i.e., bidirected edges), which means that they have the opposite direction from one DAG in the equivalence class to another DAG in the equivalence class. There is an edge (directed or undirected) between x and y, if and only if, the variables are conditionally dependent given S, for all possible subsets S of the remaining nodes [22].

### 2.3. Statistical Analysis

**Figure 1:**
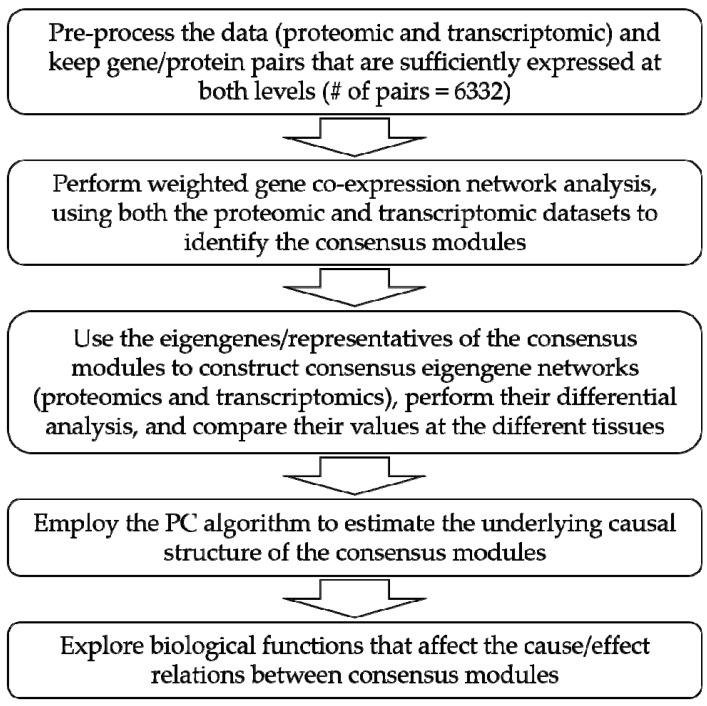
Flowchart of the analysis.

The statistical analysis was based on the protein abundances and the transcript FPKMs. It involved a stepwise approach including four main steps aiming to provide valuable biological insight in sweet cherry. These steps are briefly depicted in the first four panels of the flowchart in Figure 1.

Initially, the pre-processing of the proteogenomics data was performed as described in Xanthopoulou et al. [10]. Additionally, only gene/protein pairs with valid values for all tissues at both proteomic and transcriptomic level were selected (*n* = 7244). Of these, only the gene/protein pairs with values greater than 1 in at least 5 tissues (one out of three) at both protein and transcriptomic levels were further assessed, resulting in 6332 cases.

The next step was to use both the proteomic and transcriptomic datasets and identify clusters of gene/protein pairs at a proteogenomic level. To this end, the weighted gene co-expression network analysis, which is widely used with high-dimensional data sets for studying biological networks, was employed (with the “WGCNA” package in R) [17]. Particularly, the R function “goodSamplesGenes” was initially employed to remove unqualified genes and samples (missing entries and zero variance across the two datasets criteria apply). The function “pickSoftThreshold” was then used to select an appropriate soft-thresholding power based on the criterion of approximate scale-free topology. Then, the “blockwiseConsensusModules” function was applied to identify the consensus modules (clusters) across the proteomic and transcriptomic datasets. In all cases, the minimum module size was set to 30, the module detection sensitivity was set to 2, and the cut height for merging of modules to 0.25.

Next, based on the consensus modules detected, the “plotEigengeneNetworks” R function was employed to develop consensus eigengene network heatmaps and visualize the inter-module relationships (adjacency/correlation). These eigengene network heatmaps use the relations between the consensus eigengenes, which are basically representatives of the consensus modules, and defined as the first principal component of the expression matrix of the corresponding module. Plots depicting the pairwise preservation measures between the consensus eigengene networks heatmaps in the proteomic and transcriptomic datasets were constructed as well. Furthermore, heatmaps depicting the values of the consensus eigengenes at the tissues of interest were constructed using the “pheatmap” function. For these heatmaps, hierarchical clustering was performed for the consensus modules, using the Euclidean distance measure and the complete clustering method.

Then, the constrained-based PC algorithm was employed with the R package “pcalg” [22] to produce an estimate of the underlying causal structure among the consensus modules, using their representative eigengenes. In particular, the “pc” function was used to estimate the equivalence class of a directed acyclic graph from observational data, under the Markov assumption that the distribution of the observed variables is faithful to a DAG [22]. Since all eigengenes (variables) were continuous, the function “gaussCItest” was used to compute the conditional independence tests. The required corresponding sufficient statistic consisted of the correlation matrix of the consensus eigengenes, and their sample size. For the visualization of the resulting causal structure, the R packages “dagitty” [23], and “ggplot2” were employed [24]. The standard Pearson correlation coefficient was used as well to pairwisely assess the linear relation among the different modules (represented by the corresponding eigengenes). The analysis was performed with R Version 4.1.0 [25].

## 3. Results and Discussion

Sweet cherries are highly appreciated fruits for their taste, color, nutritional value, and beneficial health effects [11,12,13]. Although a large number of studies have been conducted to better understand sweet cherry ripening and quality, no causal-based proteogenomic information is available regarding the fruit ripening up to now. Here, we initially investigated the protein accumulation and gene expression interaction model in 15 samples covering key important tissues, including leaves, shoot, bud, flowers, stem/pedicle and fruit, of the cherry tree. In addition, we further examined fruit and stem tissue-specific causal models in respect to their proteogenomic profile, which is known to interfere with the cherry ripening process [10]. This experimental approach provides a better understanding of sweet cherry ripening at the molecular levels, which will help to improve fruit quality traits.

### 3.1. Causal Model-Based Network of Co-Expression Proteogenomic Modules in 15 Sweet Cherry Tissues

**Figure 2:**
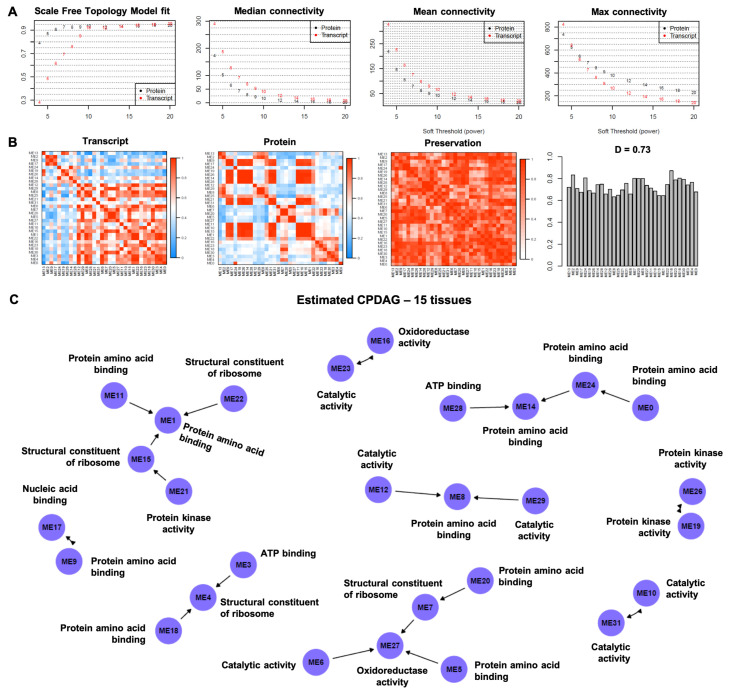
(**A**). Summary network indices are displayed as functions of the soft threshold power. The plots indicate that approximate scale-free topology is achieved around the soft-threshold power of 9. (**B**). Consensus eigengene networks are displayed and their differential analysis, based on all the 15 tissues considered (leaves, shoot, bud, flowers, stem/pedicle, and fruit). The first two plots show the eigengene network heatmaps in the proteomic and transcriptomic datasets (labeled Transcript and Protein). In the heatmaps, the red color represents high adjacency between modules (i.e., positive correlation), and the blue color denotes low adjacency (i.e., negative correlation) between the corresponding modules. The preservation heatmap (3rd plot) shows the preservation network, defined as one minus the absolute difference of the two eigengene networks. The barplot (4th plot) shows the mean preservation of adjacency for each of the eigengenes to all other eigengenes (column means of the preservation heatmap). (**C**). The completed partially directed acyclic graph (CPDAG) is displayed. Both directed and bidirected edges are present in the causal graph. The description corresponding to the modules is based on the most frequent gene ontology term, observed within each module.

The proteogenomics data from the 15 sweet cherry tissues created 6332 mRNA-protein pairs that were expressed/accumulated in both RNA and protein datasets. The correlation between RNA and protein was measured [10] within each pair with the Spearman correlation. It was found to be overall positive, exhibiting a mean correlation value of 0.23 with more than 75% positive correlations (4796 out of 6332). All gene/protein pairs were assessed and qualified for the weighted gene co-expression network analysis (WGCNA). The soft-thresholding power was set to 9 based on the scale-free topology criterion (Figure 2A). WGCNA resulted in a network consisting of 32 modules (MEs; ME0-ME31). Modules 1–31 ranged in size from 52 to 716 transcript–protein pairs (see Supplementary Table S1). Module 0 (size 1404) consisted of pairs that were outside of the other 31 modules.

The consensus eigengene network heatmaps in proteomics data (Figure 2B) showed that the modules appeared to cluster within several small blocks, e.g., modules ME11, ME10, ME15, ME1, and ME22 constitute a block and exhibit high pairwise adjacencies. On the other hand, with the transcriptomics data, the modules clustered in two small clusters (top left), and a very large cluster in the bottom right of the transcriptomics heatmap.

The causal structure of the inter-consensus module relations is depicted in Figure 2C. Both directed and bidirected edges are present in the estimated causal graph. The directed edges depict both the presence and direction of direct causal effects. The bidirected edges represent the undetermined direction, i.e., they have an opposite direction from one DAG in the equivalence class to another DAG in the equivalence class. Four pairs of modules were connected via bidirected edges (ME16 <--> ME23, ME19 <--> ME26, ME31 <--> ME10, and ME9 <--> ME17). The number of directed edges was 15, connecting 20 modules in total within five subgraphs. Five modules constitute the direct or indirect effect of other modules, without being the cause of another module (V-structure), specifically modules ME27 (directly caused by ME5, ME6, ME7 and indirectly by ME20), ME1 (directly caused by ME11, ME15, ME22 and indirectly by ME21), ME14 (directly caused by ME24, and ME28), ME8 (directly caused by ME12 and ME29), and ME4 (directly caused by ME3, and ME18).

To retrieve the functional biological processes of 15 sweet cherry tissues, we performed pathway analysis and Gene Ontology (GO) enrichment analysis and annotated highly significant terms to a causal model-based module network (Figure 2C). Gene/protein lists and gene functional descriptions of all 32 modules are presented in Supplementary Table S2. Noticeably, several modules with similar expression patterns were enriched in the same GO terms. For instance, the genes in modules ME1, ME5, ME8, ME9, ME11, ME14, ME18, ME20 and ME24 were enriched in “protein amino acid binding” terms. Closely clustered modules ME3, ME28 and ME30 were enriched in “ATP binding”, while modules ME6, ME10, ME12 ME23, ME29 and ME31 were enriched in “catalytic activity” terms. Additionally, modules ME13, ME19, ME25 and ME 26 were enriched in “protein kinase activity” terms, whereas two modules with relative far distributions (ME17 and ME27) were enriched in “nucleotide binding” terms. The majority of genes enriched in ME16 are involved in developmental regulation processes, supporting the importance of these genes and the associated processes in the developmental stages. Genes in ME0 showed GO term enrichment in organism developmental processes while genes in ME3 depicted GO term enrichment in calmodulin binding.

### 3.2. Causal Model-Based Network of Co-Expression Proteogenomic Modules across Various Sweet Cherry Fruit and Stem Developmental Stages

In a second scenario, we considered only the eight tissues that refer to the sweet cherry fruit and stem developmental stages (FS1–FS4 and SS1–SS4) in order to identify putative hub genes/proteins that are involved in the molecular mechanism of fruit ripening.

All gene/protein pairs were assessed for eligibility in the weighted gene co-expression network analysis. There were four genes/proteins in total that did not qualify for the WGCNA analysis and were excluded from further assessment, thus reducing the total number of genes/proteins to 6328. The soft-thresholding power was set to 16, based on the scale-free topology criterion (data not shown). WGCNA resulted in a network consisting of 37 modules (MEs; ME0–ME36). Modules 1–36 ranged in size from 50 to 931 transcript-protein pairs (see Supplementary Table S1). Module 0 (size 771) consisted of pairs that were outside of all the 36 other modules.

**Figure 3:**
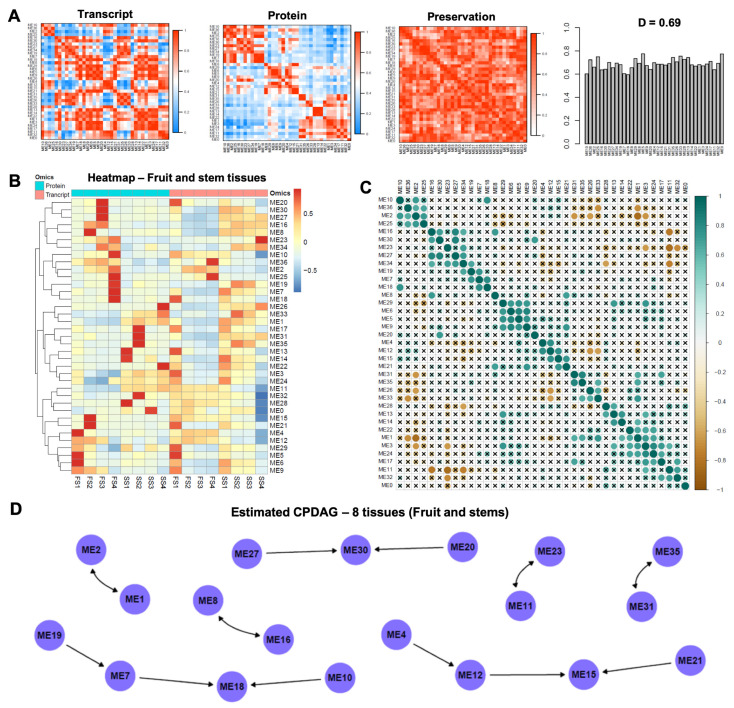
(**A**). Consensus eigengene networks are displayed and their differential analysis, based on the 8 tissues considered (FS1–FS4 and SS1–SS4). The first two plots show the eigengene networks heatmaps in the proteomic and transcriptomic datasets (labeled Transcript and Protein). In the heatmaps, the red color represents high adjacency between modules (i.e., positive correlation), and the blue color denotes low adjacency (i.e., negative correlation) between the corresponding modules. The preservation heatmap (3rd plot) shows the preservation network, defined as one minus the absolute difference of the two eigengene networks. The barplot (4th plot) shows the mean preservation of adjacency for each of the eigengenes to all other eigengenes (column means of the preservation heatmap). (**B**). Heatmap depicting the values of the consensus eigengenes (representing the consensus modules) at the 8 tissues considered (FS1–FS4 and SS1–SS4). The eigenegenes were clustered (by row) with hierarchical clustering. The distance measure used was the Euclidean distance and the clustering method was “complete”. The columns (tissues) were given in the same sequential order for the proteomic and transcriptomic (no clustering was performed by column). (**C**). Global correlation analysis (Pearson coefficient) for the 36 modules. The magnitude of the correlation is depicted in both the color and size of the spheres. Correlations which were not statistically significant at the 0.01 level were marked with an “x”. (**D**). The completed partially directed acyclic graph (CPDAG) is displayed. Both directed and bidirected edges are present in the causal graph.

The consensus eigengene network heatmaps (Figure 3A) revealed that with the proteomics data, most of the modules appear to cluster within three large blocks, e.g., modules ME10, ME36, ME2, ME25, ME16, ME30, ME23, ME27, ME34, ME19, ME7, and ME18 exhibit high pairwise adjacencies and form a large block on the top left of the proteomics heatmap. On the other hand, with the transcriptomics data, modules cluster in smaller clusters, and additionally modules within a cluster may exhibit high adjacency with modules within another cluster, e.g., ME13, ME14, ME22, ME1, ME3, ME24, and ME17 form a small high adjacency block on the bottom right of the transcriptomics heatmap, and at the same time exhibit high adjacency values with the block consisting of the modules ME7, ME18, ME8, ME29, ME6, ME5, ME9, and ME20.

In the heatmap involving the values of the consensus eigengenes at the eight tissues of interest (Figure 3B), the hierarchical clustering performed for the consensus modules revealed four main clusters. In Figure 3C, the Pearson-based correlation between the consensus eigengenes of the modules are displayed, revealing, among else, small blocks of modules that are positively linearly correlated (e.g., ME29, ME6, ME5, and ME9).

The causal structure of the inter-consensus module relations is depicted in Figure 3D. Both directed and bidirected edges are present in the estimated causal graph. Four pairs of modules were connected via bidirected edges (ME11 <--> ME23, ME1 <--> ME2, ME31 <--> ME35, ME8 <--> ME16). The number of directed edges was eight, connecting in total eleven modules within three subgraphs. Three modules constitute the direct or indirect effect of other modules, without being the cause of another module (V-structure). Particularly, modules ME15 (directly caused by ME21, ME12 and indirectly by ME4), ME18 (directly caused by ME10, ME7 and indirectly by ME19), and ME30 (directly caused by ME20, and ME27).

**Figure 4:**
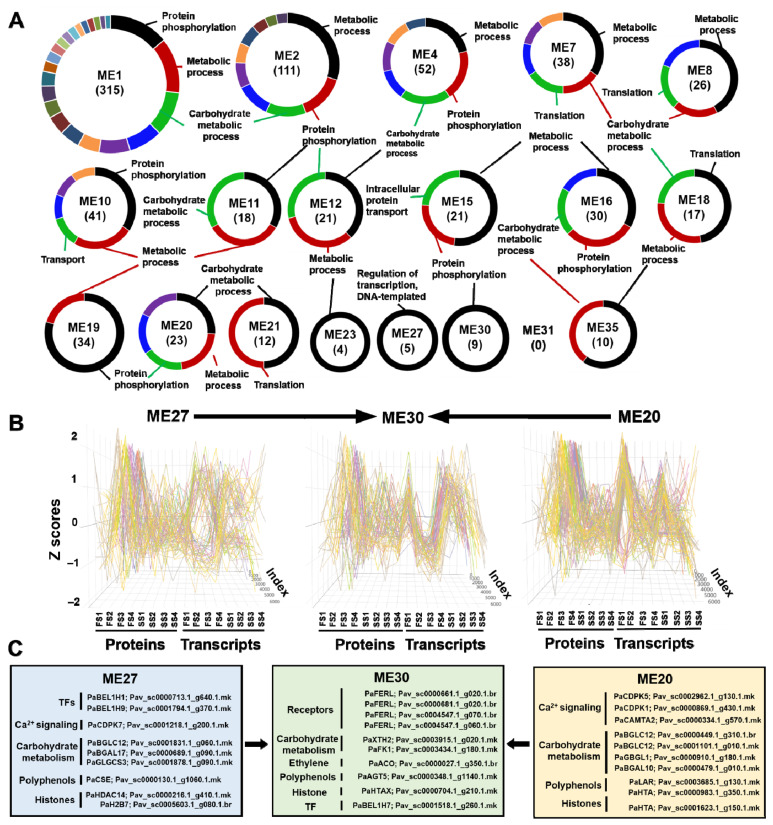
(**A**). Gene ontology (GO) categories regarding biological processes (BP) of 19 modules that were displayed in at least four transcripts/proteins. Parentheses indicate the number of unique genes/proteins that are classified in BP-GO. (**B**). Z-score trend lines of proteins and transcripts across four fruit (FS) and stem (SS) stages in modules ME27, ME30, and ME20. (**C**). Specific genes/proteins of ME27, ME30, and ME20 that are classified in ripening related groups. Arrows indicate direct effects between modules. Data provided in Supplementary Tables S3 and S4.

By focusing on the identified modules by WGCNA analysis that were included in the causal graph, three main gene ontology (GO) categories were found in the intra-module that dominated based on the biological process of each gene or protein. These categories included (i) protein phosphorylation, (ii) metabolic process, and (iii) carbohydrate metabolic process (Figure 4A). Of particular biological interest was the direct causal effect between modules, especially when a V-structure scheme was observed. The focus was on ME30 that was directly caused by ME20 and ME27 and their relations across the various sweet cherry fruit development stages.

Initially, the gene/protein pairs exhibited similar patterns within each of the three modules (ME30, ME20, and ME27), based on z-scores in proteins and transcripts across the fruit and stem developmental stages (Figure 4B). On top of that, these patterns were distinct among the three modules. To further analyze the ME30 concerning the direct causal relationship with the ME20 and ME27, genes/proteins that may have caused this direct effect were targeted (Figure 4C).

Based on the literature [26,27,28,29,30,31,32,33,34,35], these observed direct cause-effect relations can be justified. For instance, the Ca^2+^ signaling category of ME20 (PaCDPK7; Pav_sc0001218.1_g200.1.mk) and ME27 (PaCDPK5; Pav_sc0002962.1_g130.1.mk, PaCDPK1; Pav_sc0000869.1_g430.1.mk, PaCAMTA2; Pav_sc0000334.1_g570.1.mk) may be linked to the synthesis of phenylpropanoids, notably anthocyanins during fruit ripening (ME30, PaAGT5; Pav_sc0000348.1_g1140.1.mk) [26]. In addition, caffeoyl shikimate esterase of ME27 (PaCSE; Pav_sc0000130.1_g1060.1.mk), which is included in phenylpropanoid biosynthesis, participates in the process of the endocarp lignification in stone fruits [27]. Moreover, leucoanthocyanidin reductase of ME20 (PaLAR; Pav_sc0003685.1_g130.1.mk) has been found to increase when gibberellic acid (GA_3_) was exogenously applied in sweet cherries during fruit development [28]. This protein was associated with delayed fruit coloring due to inhibition of anthocyanin biosynthesis in sweet cherry [28] and apple [29] fruit.

Another interesting biological example regarding the function of the identified modules referred to the fruit softening process. It is known that sweet cherry fruit softening results in changes in cell wall structure and composition due to cell-wall-modifying enzymes [30]. Herein, several cell wall-related enzymes, such as PaBGLC12; Pav_sc0000449.1_g310.1.br, PaBGLC12; Pav_sc0001101.1_g010.1.mk, PaGBGL1; Pav_sc0000910.1_g180.1.mk of ME20, and PaBGLC12; Pav_sc0001831.1_g060.1.mk of ME27, are directly involved in the degradation of cellulose through splitting the cellulose chain into cellobiose and glucose or cellobiose into glucose [30]. Furthermore, the identified β-galactosidase enzymes of ME20 (PaBGAL10; Pav_sc0000479.1_g010.1.mk) and ME27 (PaBGAL17; Pav_sc0000689.1_g090.1.mk) are involved in cell wall degradation of pectin and hemicellulose [30]. Probably, cell wall loosening-related protein activities that were found in ME20 and ME27 may affect PaXTH2; Pav_sc0003915.1_g020.1.mk of ME30 through cleaving primary cell wall xyloglucan polymers. It was recently shown that PaXTH2 is tightly linked to ethylene production in apples via activation of PaACO; Pav_sc0000027.1_g350.1.br (ME30) resulting in fruit softening [30]. In parallel, starch biosynthesis enzymes of ME27, such as PaGLGCS3; Pav_sc0001878.1_g090.1.mk [31,32] may act at PaFK1; Pav_sc0003434.1_g180.1.mk of ME30, since it was found that it regulates plastid differentiation into chloroplasts, instead of storage plastids, such as amyloplast [33,34].

The current analytical approach provided further information about fruit and stem development that has not been clarified until now. A characteristic paradigm was the transcription factors (TFs) PaBEL1H1; Pav_sc0000713.1_g640.1.mk and PaBEL1H9; Pav_sc0001794.1_g370.1.mk of ME27 that regulate chloroplast development and chlorophyll synthesis [35]. These TFs may influence PaBEL1H7; Pav_sc0001518.1_g260.1.mk of ME30 (Figure 4C) that were found to be involved in the chlorophyll degradation via ethylene production during fruit ripening [36]. The ME30 was characterized by the presence of FERONIA receptors since four of them (PaFERL; Pav_sc0000661.1_g020.1.br; Pav_sc0000681.1_g020.1.br; Pav_sc0004547.1_g070.1.br; Pav_sc0004547.1_g060.1.br) were contained in this module. It has been suggested that Feronia receptor kinases modulate multiple signaling pathways through phytohormone regulation across fruit development [37], indicating a possible module for causal reactions into the ripening process (Figure 4C). Previous studies also documented that post-translational modifications of histones influence chromatin organization and contribute to the epigenetic regulation of gene expression during fruit development and ripening [38,39]. The analysis herein has shown that PaHDAC14; Pav_sc0000216.1_g410.1.mk and PaH2B7; Pav_sc0005603.1_g080.1.br of ME27 and PaHTA; Pav_sc0000983.1_g350.1.mk; Pav_sc0001623.1_g150.1.mk of ME20 directly affected PaHTAX; Pav_sc0000704.1_g210.1.mk of ME30 (Figure 4C), showing that sweet cherry fruit ripening was accompanied by chromatin reprogramming and epigenetic modification.

## 4. Conclusions

Causal discovery may boost molecular data exploration and the characterization of key biological processes in plants. Herein, using causal models in a large proteogenomic data set obtained from different sweet cherry tissues, cause–effect relations between the consensus gene/protein modules were uncovered that reflect important biological functions related to the ripening process. These results could be used as a reference for either experimental validation (e.g., functional analysis in other species) and/or to set future biological questions. The application of causal models in the proteogenomic era might be combined with all available modern molecular tool practices, such as bioinformatics and innovative decision-making systems, to create novel approaches in fruit tree biology and cultivation.

## Data Availability

The database used is available (www.GrCherrydb.com, accessed on 1 November 2021) to other researchers for purposes of reproducing the results. The data presented in this study are available on request from the corresponding authors.

## Supplementary Materials

The following are available online at https://www.mdpi.com/article/10.3390/cells11010092/s1, Table S1: Size of modules identified by weighted gene co-expression network analysis (WGCNA) across (a) all tissues, and (b) fruit and stem, Table S2: Information related to modules with causal relations identified by weighted gene co-expression network analysis (WGCNA) across all tissues, Table S3: Information related to modules with causal relations identified by weighted gene co-expression network analysis (WGCNA) across fruits and stems, Table S4: Module classification and selected genes/proteins across fruits and stems.
